# Thomas Sackville's Hall of Fame: displaced, reinvented and preserved at Knole

**DOI:** 10.1098/rsnr.2021.0044

**Published:** 2022-06-20

**Authors:** Olivia Stoddart, Gerry Alabone

**Affiliations:** ^1^ The National Gallery, Trafalgar Square, London WC2N 5DN, UK; ^2^ The National Trust, Heelis, Kemble Drive, Swindon SN2 2NA, UK; ^3^ Department of Art History, Curating and Visual Studies, Barber Institute, University of Birmingham, Edgbaston, Birmingham B15 2TT, UK

**Keywords:** Knole, Thomas Sackville, Cartoon Gallery, portrait set, Brown Gallery, Francis Parsons

## Abstract

Between 1605 and 1608 Knole was transformed into a dazzling Renaissance palace by Thomas Sackville, 1st Earl of Dorset. All around its largest Gallery ran a frieze of nearly fifty oval portraits, of which thirty-eight survive on their original rectangular framed oak panels. In 1702 the paintings were prised from their walls and moved elsewhere in the house. The surviving panels had deteriorated, mostly owing to movement in their wood being restrained by their original engaged batten frames. Sources state that in 1793 the paintings restorer Francis Parsons was responsible for significant interventions that effectively changed the set from fragments of a fitted Jacobean decorative interior into a hang of individual paintings in a more contemporary neoclassical livery. Aesthetic changes made by Parsons reflect a change in taste during this era whilst simultaneously addressing the paintings' physical deterioration—principally interventions to the back of the panels in an attempt to keep the portraits flat. Additionally, the set was augmented with six portraits, possibly older than Sackville's original set. These treatments carried out during the eighteenth century to stabilize, restore, augment and update this important Jacobean portrait set demonstrate careful manipulation of their condition and significance.

## Introduction

**Figure 1.**
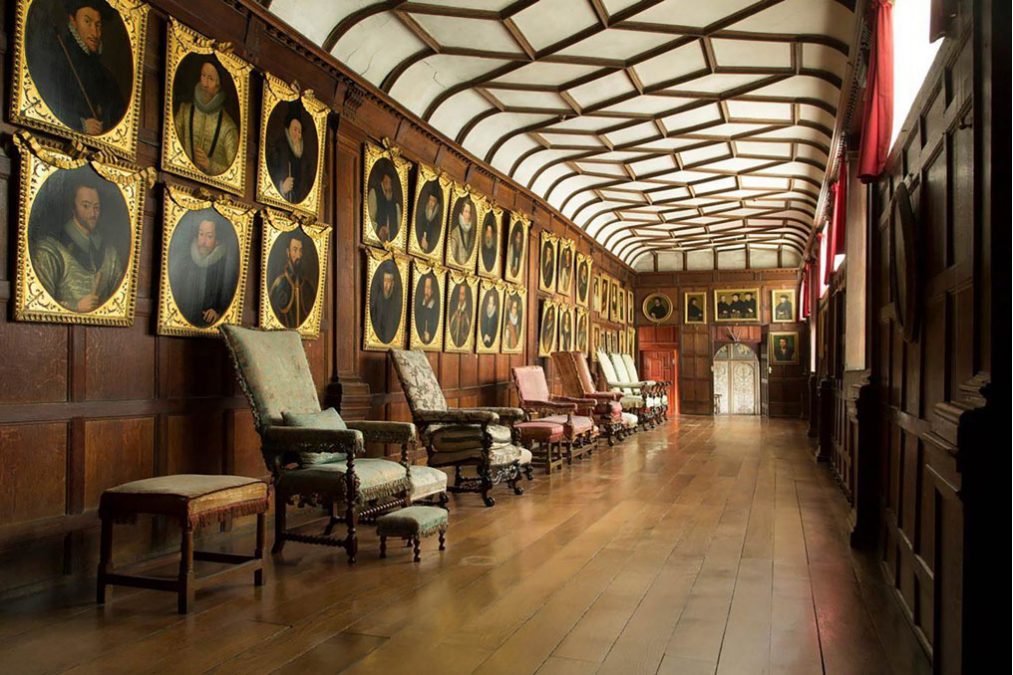
The portrait set displayed in the Brown Gallery, Knole, 2013. Copyright © National Trust/John Miller. (Online version in colour.)

**Figure 2.**
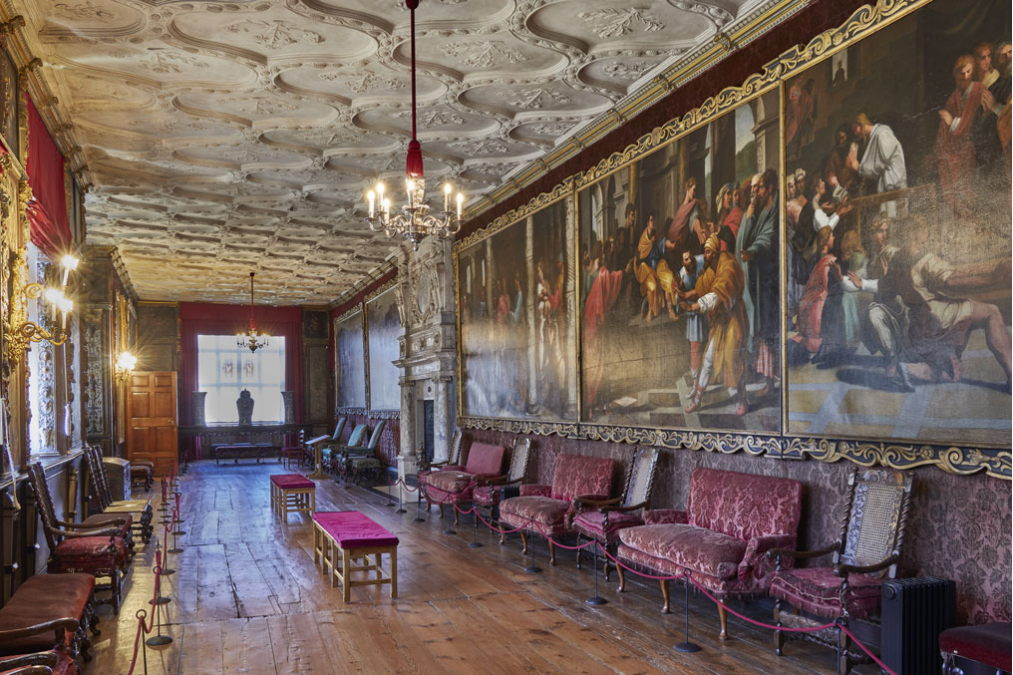
The Cartoon Gallery, Knole, looking west, 2018. Copyright © National Trust/Laurence Pordes. (Online version in colour.)

This paper considers an important Jacobean portrait set (referred to here as the Knole portrait set), which has remained in the house for which it was made but has undergone significant treatments over the last three centuries to conserve, restore and augment its material condition and display context. The set now comprises forty-four matching portraits of English monarchs, noblemen, courtiers, clergy, and naval and military commanders from the Tudor and Jacobean periods. The portraits are based on standard types and are thought to have been painted in England; however, research has not yet been able to determine the artist, or artists, who made them. The set has been displayed in the Brown Gallery at Knole since the eighteenth century (figure 1). However, doctoral research by Catherine Daunt^1^ and dendrochronological analysis by Ian Tyers^2^ have confirmed that the set was made between 1607 and 1608 for its owner Thomas Sackville, 1st Earl of Dorset (1536–1608). The set originally formed an integral part of the decorative wall panelling of a different Gallery at Knole, now known as the Cartoon Gallery. This Gallery was itself the culmination of a processional route leading to the King's Bedroom prepared by Sackville in the hopes of a visit from James VI & I. Sackville remodelled the former hunting lodge into a dazzling Renaissance palace, and his showrooms survive, variously altered (figure 2). Portrait sets in the Elizabethan and Jacobean eras served as a tool to communicate the patron's political ideologies. As prints and paintings came into wider circulation during this period, the development of recognizable portrait types created a clear way for Sackville to show visitors to Knole his connections and aspirations.^3^ Daunt states how all the portraits from the original set are derived from painted or printed prototypes extant before 1608,^4^ and many were standard figures in late Tudor and Jacobean portrait collections. For example, many of the figures represented in the Knole set were also included in the set made for Weston Park (Warwickshire) around fifteen years before.^5^ This and other such sets typically included royal portraits. However, the Knole set is particularly remarkable as most of the portraits survive within the original house—if not their actual room setting.

A description from a former steward at Knole published in 1817 recalls that the portraits hung below the cornice in the polychrome frieze of the Cartoon Gallery and were removed in 1702 when copies of the Raphael cartoons^6^ were brought from Copt Hall. The cartoons were hung on the north and southwest walls, where they remain to this day.^7^ The spaces on the west and eastern south walls that originally held portraits are now occupied by flower paintings, which are thought to have been added in 1696.^8^ Daunt established that six of the portraits, including all four depicting monarchs, had been introduced into the set by 1793 when the painting restorer Francis Parsons gilded and painted the spandrels for all the portraits and added carved ribbons with inscriptions, as recorded in a bill.^9^ The work by Parsons was done under the patronage of John Sackville, 3rd Duke of Dorset (1745–1799), who was also a prolific collector of Old Master paintings and supporter of contemporary artists practising in England.

Building upon a technical study undertaken by Ruth Jongsma,^10^ recent material and technical analysis by the present authors and others referred to in this article has established much of the original arrangement and decoration of the paintings and panelling. Though the portrait set formed an integral part of Sackville's intellectual and political ambitions for the house's interior,^11^ It underwent significant changes following removal from the original Gallery just under 100 years after being made. Attempting to understand changes resulting from deterioration, treatment and augmentation helps us to comprehend the tastes and ambitions of later members of the Sackville (later Sackville-West) family at Knole, and what actions were made towards those ends.

Knole's showrooms have been accessible to the public since the eighteenth century and the house was given to the National Trust in 1946. The ongoing scholarly interest in the house and its need of remedial conservation culminated in the ‘Inspired by Knole’ project between 2015 and 2020, which restored the roof and conserved much of the interiors and collections. Recent research conducted at Knole has been actively cross-disciplinary, in addition to scholarship provided by dissertations and publications considering the house's functional and architectural transformations.^12^ This paper's authors were involved in the project—Gerry Alabone treated frames and furniture from the house as well as carrying out material and technical research of the interior of the Cartoon Gallery and the portrait set's framed panels—and Olivia Stoddart conducted a technical study of the portrait set as part of her postgraduate thesis at The Courtauld Institute of Art.^13^

The scope of this paper is the portrait set's treatment and presentation between the early eighteenth and twentieth centuries and it considers the following questions: how successful were the treatments to the objects' structural and decorative condition, in terms of intended past and present-day approaches; and what do the treatments reveal about the significance of the objects' changing display contexts? Both questions are addressed in the following sections: their original display context in the Cartoon Gallery and removal; subsequent relocation and deterioration; major restoration by Francis Parsons; the addition of four royal portraits to the set, and later changes. A material and technical approach to the objects and their setting is used in this paper in conjunction with documentary evidence.

## Original display context and removal

The portraits were commissioned by Thomas Sackville near the end of his life to compose a ‘Hall of Fame’. The portraits alternated with carved polychrome term figures to form a frieze completely circuiting the Gallery above its painted oak panelling and wall hangings. This was also originally the arrangement at Weston Park.^14^ The decorative scheme surrounding the paintings is thought to have been created in part by Paul Isaacson.^15^ Most of the term figures survive at Knole although the majority have been removed from the Gallery. Each figure has different features, including the trophies adorning their pillar bases. These can be interpreted allegorically; however, as the terms' physical relationship to individual portraits in the set is mostly lost, specific interpretations intended by their design has also been broken by their deinstallation.

**Figure 3.**
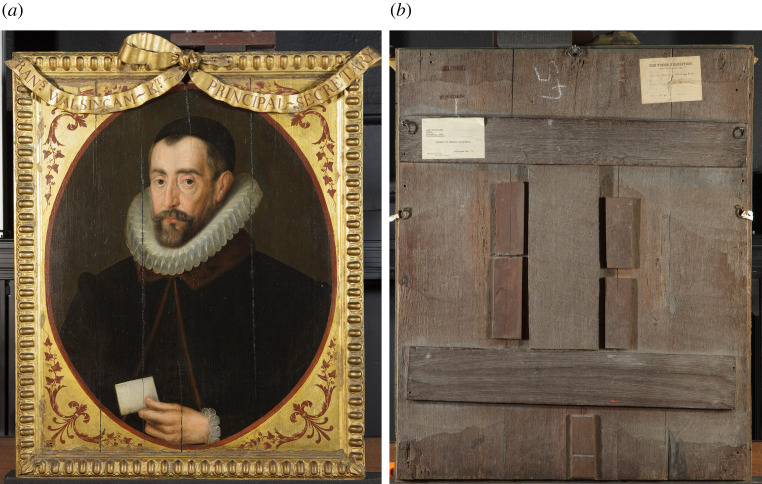
Front (*a*) and back (*b*) of the portrait of Sir Francis Walsingham (1530–1590), after John de Critz, British (English) School, 1605–1608 and 1793 (repainted and embellished frame), painting 129771 and frame 131182, 850 × 700 × 60 mm. Copyright © National Trust/Olivia Stoddart. (Online version in colour.)

**Figure 4.**
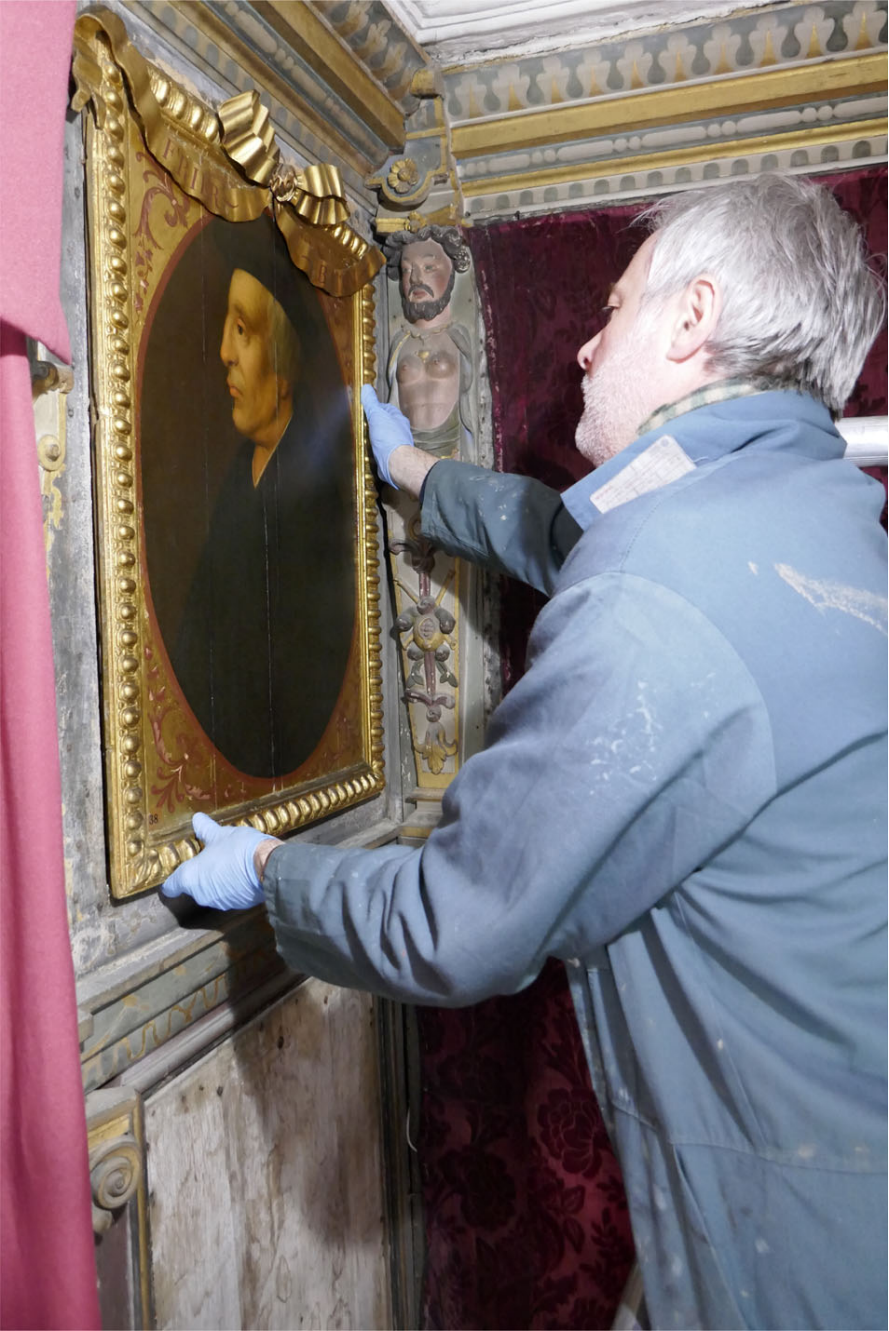
Northwest corner of the Cartoon Gallery with painting being temporarily replaced in the frieze, 2019. Portrait of Friar Roger Bacon (*ca* 1214–*ca* 1292), British (English) School, 1605–1608 and 1793 (repainted and embellished frame), painting 129764 and frame 131175, 830 × 620 × 70 mm. Copyright © National Trust/Daphne Gilbert. (Online version in colour.)

The portraits were painted in oil onto tongue-and-groove joined oak panels made from between two and six vertically aligned eastern-Baltic oak boards (figure 3). The construction of the panels has been dated by dendrochronology to around 1605.^16^ Each had an engaged frame comprising four oak battens on the front, attached by approximately twelve clenched rose nails driven in from the back. The portraits were fixed into their recesses in the frieze by nails driven through the edges of the frames from the front. All of the portraits are thirty-two inches high (813 mm), including the frame, but their widths vary according to the particular wall within the Gallery for which they were made. Sample analysis has confirmed that the carved frames were added to the panels prior to the application of the preparation and paint layers.^17^ Significantly, the top and bottom frame members were nailed across the grain of the panels, which has subsequently produced many splits owing to the panels' contraction being resisted by their frames. During the ‘Inspired by Knole’ project, templates were made of the nail holes around the frames to match them to their recesses in the surviving walls of the Cartoon Gallery (figure 4), thereby establishing their original order of hang.

The original decorative scheme in the panels' frames and spandrels had a similar format to what can be seen today—an oval line separating the painted portraits and spandrels, and inscriptions.^18^ However, their colour and gilding have been changed considerably. The oval line is thought to have been originally oil gilded—which is seen in several paintings, from photomicrographs of areas of lifting paint in the upper layers, and a cross-section taken from the portrait of *Bourbon*.^19^ Seven portraits were X-rayed, and, of these, three show evidence of inscriptions of the sitters' names and titles beneath the current gilding in the spandrels.^20^ In *Bourbon* and *Burghley*^21^ the letters are dark in the X-ray, suggesting a material that is transparent to X-rays. Yet, in *Walsingham*^22^ the letters are light, suggesting an X-ray-absorbing material, possibly lead- or gold-based. These inscriptions are thought to relate to the original decorative layers. Although only three paintings have evidence of inscriptions, it seems likely that this was a common feature across the entire set. Further research would help to confirm this. Analysis of the inscription in the portrait of *Thomas Wolsey*^23^ (now in the National Portrait Gallery (NPG), London, but originally forming part of the Weston set) showed that the lettering consisted of a lead–tin yellow paint and gilding (or gilded highlights) on top.^24^ In the Knole portraits there are glimpses of black seen through losses to the upper campaign(s) and several cross-sections suggest that black may have been a large part of the original spandrel design. If indeed the inscriptions were gold/yellow in colour, set against a black background, this contrast would make for good legibility when viewed from a distance. This proposed scheme would incidentally match the colours in the surrounding frieze (black and gold; figure 4).^25^

**Figure 5.**
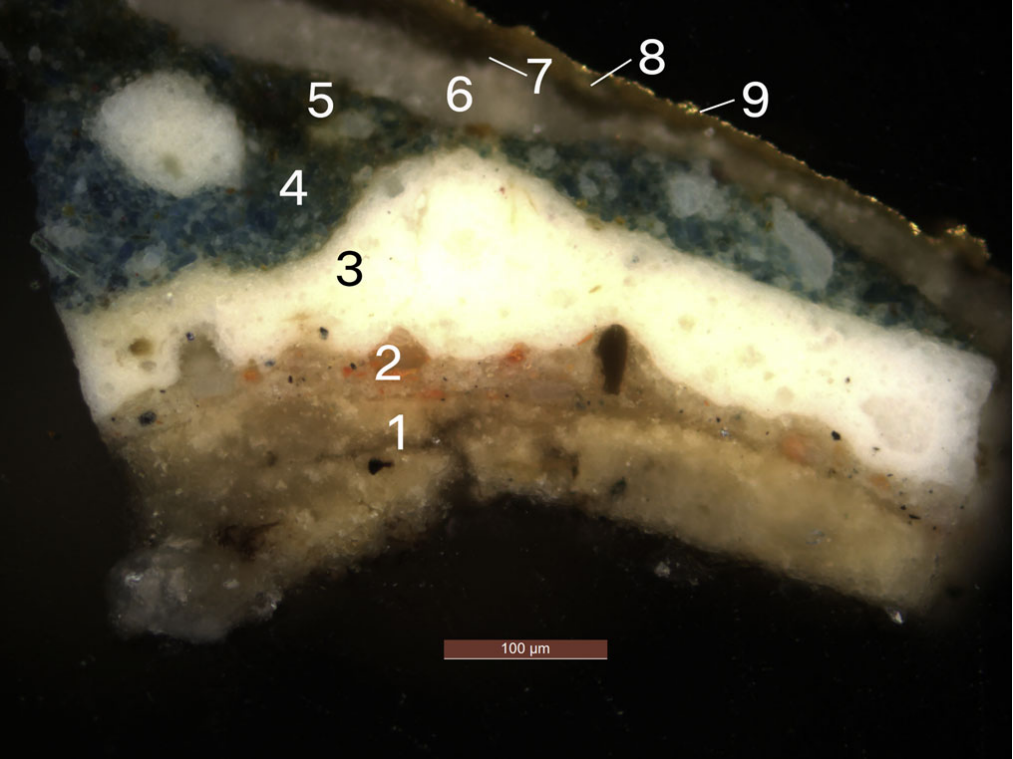
Build-up of layers in frame, 2019. Copyright © National Trust/Olivia Stoddart. This sample was taken from the carved rib ornament on the frame of Charles Bourbon. Two campaigns are present in this section of the frame: first, an azurite-containing layer thought to be original; second, Parsons' campaign from 1793. The elemental composition of each layer was inferred from scanning electron microscopy–energy dispersive X-ray analysis (SEM-EDX). (1) Chalk ground. (2) Lead white, red lead and carbon black priming. (3) Lead white. (4) Azurite and lead white. (5) Azurite and lead white (less lead). (6) Chalk. (7) Organic layer. (8) Yellow oil mordant. (9) Gold leaf. (Online version in colour.)

The mouldings of the engaged frames, carved with ribbed strapwork, were found originally to have had gilded and azurite painted areas. A photomicrograph taken of the strapwork of the frame of *Howard*^26^ shows a blue layer beneath the flaking gilding of the later upper scheme. A cross-section from *Bourbon* shows this layer (figure 5: layers 4 and 5); SEM-EDX^27^ analysis suggests that it is the blue copper-containing pigment azurite. A different cross-section taken from the rib of the same frame shows a layer of gilding beneath the later upper scheme. These two samples suggest that the ribbed strapwork was originally an alternating pattern of painted azurite and gilding. The blue, gold and black scheme described for the frames and spandrels matches the colours seen in the original frieze in the Cartoon Gallery and other decorative elements of the room (figure 4).^28^ X-ray fluorescence spectroscopy (XRF)^29^ analysis of other paintings also suggests that the frames were painted azurite with gilded ribs, sight edge and back edge.^30^ Therefore, when it was first created the portrait set's value was in keeping with the whole decorative interior, which was richly carved, painted and gilded with sophisticated allegorical figures and motifs.

Material evidence from the Cartoon Gallery's surviving panelling and brackets in the cornice indicates there were spaces for approximately fifty portraits in the frieze around the four walls,^31^ so it is likely that about ten from the original set are now lost. At least six of these were probably originally displayed along the eastern south wall of the Cartoon Gallery—strongly suggested by the fact that all of the surviving portraits have been matched to specific recesses in the other surviving walls by physical evidence. All of the frieze from this section of the eastern south wall has been replaced in the past, and the vestige of surviving panelling in this wall has historically been heavily infested with woodworm and damaged by damp from the six windows. The room was heated from one huge fireplace some distance away, which, together with the large windows, would have created an environment of fluctuating temperature and relative humidity.^32^ In response to these changes the wooden panels would have expanded and contracted, culminating in the formation of splits because of their inability to move unimpeded across the grain owing to their engaged frames. It seems reasonable to conclude that the lost portraits along the eastern south wall were also extensively damaged by woodworm, and that several were selectively disposed of some time after being taken down in 1702. Therefore, material evidence indicates that the panels deteriorated in this Gallery, being variously damp and susceptible to infestation, or too dry, causing splitting due to shrinkage. When they were prised off the Gallery's frieze, the nails fixing them to the walls through the edges of their engaged frames also caused significant localized damage and loss. The sitters of the lost portraits are unknown; however, as the other portraits are grouped by sitter category (i.e. clergy, statesmen, foreign figures), and as the lost portraits would have hung over windows facing paintings of lesser English nobility, this suggests that they were not of the highest social status.

The Cartoon Gallery's rich and complex original interior was sacrificed to make room for a set of six high-status paintings from Copt Hall. The portrait set was removed from the walls along with most of their adjoining term figures and were stored or redisplayed elsewhere in the house. The empty frieze and walls below were covered with a unifying wall fabric on which to hang the Cartoons. The portrait set's removal and redisplay elsewhere in the house may indicate that the significance of the original context of the Gallery's interior scheme had become less important than the paintings themselves by the early eighteenth century. The impact of the set's removal from the Cartoon Gallery on their structural and decorative condition is mixed, beyond that of the physical damage caused by deinstallation. As a direct result, the additional freedom of movement led to their present, typically twisted alignment. Of course, their structural and decorative condition was not the reason for deinstallation and their frames would have needed to be permanently detached to reduce splitting in the panels, as is still the case. It is interesting that the removal of the frames has apparently never been considered desirable, or indeed acceptable, and the original frames remain engaged with their portraits.

## Relocation and deterioration

Throughout their 400-year history, the portrait set has been hung in several locations within Knole. Through documentary sources, several of these locations have been suggested.^33^ An inventory^34^ from Knole in 1706 records ‘Thirty two Heads’ in the Leicester Gallery and ‘21. heads’ in the passage leading to the Leicester Gallery (likely to be what is now the Brown Gallery). George Vertue (1684–1756), British antiquarian and engraver,^35^ visited Knole in 1728 and recorded thirty-seven heads in ‘a small Gallery with pictures. on bord all alike in size and ornament’.^36^ Vertue is no doubt referring to the Knole portrait set hanging in what was then known as the Horn Gallery, a section of what is now the Brown Gallery.^37^ His description suggests that by 1728 the majority of the set had been moved to the Brown Gallery; by 1799 the partition dividing the Galleries was removed, creating the present-day layout of the Brown Gallery.^38^ In 1799 an inventory listed ninety paintings in the Brown Gallery, including forty-two from the current set of extant paintings, most likely counting four of the unoriginal additions that had been integrated into the set by this time, discussed further below.^39^ It is not unprecedented in the eighteenth century for stately homes to refashion viewing spaces for the enjoyment of art.^40^ The new location for the Knole set may reflect this change in attitude towards using architecture to showcase collections, by transitioning the space into a fashionable double-hang closer to eye level rather than part of a skied frieze.

Evidence for the deterioration of the paint layers in the surviving original portraits has been gathered through several technical studies, the results of which may provide additional insight into why the set has decreased in size over the years as well as motivations for the significant restoration campaign in 1793. The fundamental structure of the engaged frames has caused many issues over the years; the campaigns on the reverse that were carried out to address these structural problems are described below. The movement of the panels and engaged frames in response to the uncontrolled environment in the house would also have caused instability in the paint layers surrounding the joins and splits in the wood.^41^ Additionally, the environment in the house would have affected the stability of the hygroscopic materials in the layer structure; notably the organic binder in the chalk ground layer would have been affected by fluctuating levels of relative humidity.^42^ It is impossible to know whether the structural and decorative condition of the set would have been better or worse had they remained in the original frieze. However, their simple survival is remarkable and demonstrates a continuity of hugely impressive ongoing care over three centuries.

There is some confusion over the smalt layer which is found always above the early decorative scheme of the spandrels; potentially it could be evidence of an intermediate restoration campaign.^43^ For example, in all samples with a complete layer structure there is a chalk-containing ground layer found beneath the smalt, suggesting a priming for a new layer. The smalt is also seen dripping into splits in the panel in the portrait of *Howard*. In some instances, the smalt layer is found directly on the wood with fragments of ground beneath it, suggesting that it is painted over damage. In a cross-section taken by Jaskierny from *Henri de Montmorency*,^44^ the blue smalt is seeping into cracks in the black paint layer beneath. There are many inconsistencies found when sampling the layer structure, which mean it is not possible to conclude whether the blue smalt was part of the original scheme or painted over an already damaged surface. The use of the pigment spans this paper's principle period of interest (*ca* 1608–1793),^45^ which could mean it was original or an intervention.^46^ It is, however, almost certain that it predates Parsons' restoration, as it underlies it. The smalt layer has been a liability to the stability of Parsons' campaign. This interface is the site of much delamination and was a focus of the most recent 2017 conservation campaign (among other issues).

## Francis Parsons' restoration

Francis Parsons (*ca* 1740–1804) was an artist, dealer and restorer practising in London in the latter part of the eighteenth century.^47^ In 1793 the 3rd Duke of Dorset commissioned Parsons to restore the Knole set; to date, this is the only known restoration project by Parsons. He had some success in portrait painting, exhibiting often at the Society of Painters, and his painting of the engineer James Brindley (1770)^48^ can be found today in the NPG. Alas, his contemporaries perhaps did not deem his attempts in portraiture a triumph. Edward Edwards suggested that ‘he became a picture dealer and cleaner—a good resource for the invalids in painting’.^49^ The milieu within which Parsons was practising appeared to blur modern boundaries between artist, dealer and restorer. Many of Parsons' contemporaries were practising artists and restorers—for example, William Seguier (1772–1843), first Keeper of the National Gallery, who initially identified as an artist before entering the profession of picture restoration.^50^

Parsons charged four guineas ‘For cleaning & Repairing forty old portraits on Pannels … and the Frames mended and new Gilt, with Ribbons added to each Frame and label'd with the name and title of each portrait, and the Angle of each painted with ornaments’.^51^ By this time, as well as the splitting, it is likely that the panels had also become warped following their removal from the Cartoon Gallery frieze.^52^ To repair the panels it therefore seems likely that Parsons temporarily removed the majority of the panels' top and bottom frame members in order to rejoin splits. Material evidence shows that the clenched nails (which attached the frame members) were removed from the back of the panels by first cutting wood from around the nail heads to enable them to be prised out. Parsons then planed the backs of the panels, making them thinner and more flexible about the centre, and added H-shaped oak battens to the backs of more than half of the panels to keep them flat (as seen in figure 3*b*). These interventions would also have been intended to reduce the chance of the panels splitting again in future. However, it does not appear that this treatment was particularly successful, as future restorers replaced some of the battens with alternative methods. Within the context of eighteenth-century painting restoration, it was not uncommon for restorers to drastically change the reverse of panels through interventions such as thinning or the adding of additional wooden supports.^53^ Past damage to the frames, including losses from their back edges caused by removal from the Cartoon Gallery frieze, were filled with a putty of chalk and collagen glue, and overgilded. Technical evidence showing that the overgilding occurred only once corroborates the material evidence that the structural work described above was carried out as one treatment by Francis Parsons.

The thinned panels are indeed more flexible, albeit weaker. However, this does not reduce their dimensional instability across the grain when reacting to changes in relative humidity. Therefore, this thinning treatment may be considered a failure, as splits and joins have continued to be unstable and have opened markedly. The attempt to keep the panels flat by the addition of battens has also been unsuccessful, with many remaining noticeably warped. It is now commonly regarded by conservators that eighteenth- and nineteenth-century practices of thinning and bracing panels through battens or cradles often exacerbated structural issues.^54^ Today conservators take a more conservative and minimal approach to treating structural issues in panels.^55^

Technical evidence suggested with more accuracy the materials Parsons used to create the decorative scheme. A small paint sample taken from the ribbed frame enabled analysis of the preparation and paint layers used. The cross-section (figure 5) shows an additional layer structure (layers 6–9) on top of the original scheme (layers 1–5). The composition of each layer was inferred by SEM-EDX for pigment identification and staining tests to determine whether the binding medium was protein- or oil-based. Layer 6 shows that a chalk-based layer was painted wholescale over the frames and spandrels to block out the existing scheme and to lay a foundation for the subsequent layers. On top of this chalk layer is a proteinaceous organic layer (its exact composition untested), an oil-based yellow mordant layer, and finally gold leaf on top. This layer structure was noted in more than one cross-section from different panels.^56^ The red foliate decoration and oval line around the portraits were later applied on top of the gilding and are thought to be painted in an earth-based pigment (identified by the presence of iron in XRF) bound in oil.^57^ In the case of the red oval lines there are some differences between the panels. Most are painted with a continuous tone (for example, *Wolsey*), and a few are painted with tonal differences in opposite corners to give a three-dimensional illusion and to enhance the suggestion that the portraits are in separate planes from the spandrels and frame (seen in the portrait of *William of Nassau, Prince of Orange*).^58^ This might suggest that more than one hand was responsible for the execution of Parsons' campaign. It is possible that Parsons was working alongside a team of skilled individuals, or could have outsourced the work to carvers and gilders, as the task of transforming the set also demanded these specialist skills. The lasting effects of Parsons' restoration intervention are varied across the set. Some paintings have fared better than others over time and, in several, serious delamination is occurring between his work and the possible smalt interlayer, as mentioned above. It has been suggested that the necessity of treating the reverse of the panels and the action of removing the clenched nails may have caused additional damage to the front of the paintings, providing another impetus for Parsons' decorative intervention.^59^

In addition to the deterioration described above, a change in taste in the latter part of the 1700s may have also motivated this restoration campaign. Although Parsons did little to alter the material of the original frame members,^60^ elements of Parsons' choice of decoration are in keeping with the style of early neoclassical framing practices. Notably, the gold applied continuously across the frames and spandrels, the painted red-leaf ornament and font type are characteristic of the period. The addition of carved bows and ribbons or festoons is a feature of many neoclassical frames from this era.

Such drastic aesthetic alterations to paintings as seen in the Knole set are not unprecedented. Although within a different context, the well-documented recent restoration history of the Ghent Altarpiece (*The Adoration of the Mystic Lamb*, Hubert and Jan van Eyck, 1432) illustrates the extent to which previous restorers took artistic licence when majorly reworking areas of paintings. In the case of the Ghent Altarpiece much of the painting was meticulously reworked in the sixteenth century; in some panels up to half of the surface has been reworked.^61^ In the seventeenth century the outer frames were overpainted in a green paint, covering original inscriptions.^62^ This demonstrates that the work of Parsons carried out in the eighteenth century can be considered as part of a longer history of restorers reworking large parts of paintings deemed damaged or stylistically outdated.

When paintings have a change of display context or are restored, sometimes their frames are replaced. However, despite significant alterations to the panels, their engaged frames survive in form and function, with overgilding and some additional painted ornament. Overgilding was a typical treatment for degraded giltwood surfaces in England, until less invasive conservation treatments became favoured during the latter twentieth century. The recent ‘Inspired by Knole’ project worked on many other frames, with sometimes multiple overgilding schemes, not carried out by Parsons.^63^ Numerous frames at Knole were also resized for use on different paintings, also common for the period, and augmented with reused or newly carved ornaments.^64^

There are few known comparative technical studies of portrait sets on this scale. Where they exist the objects are of a fundamentally different format, i.e. without fixed decorative elements. A contemporary example with major technical study is the set of English kings and queens (1590–1610) now in the NPG.^65^ Although the set's early history of display is unknown, sources reveal that in the nineteenth century the portraits hung in a Gallery at Hornby Castle and served a function not dissimilar to the Knole set during this time.^66^ Parsons' drastic alteration of the Knole set not only brought the paintings into the eighteenth-century realm, but also served as part of a longer history of repurposing decorative objects into portable easel paintings reminiscent of high art within a more contemporary canon. For example, after the Weston House set was removed from their original architectural setting and dispersed, between the late eighteenth to early nineteenth centuries, they were individually reframed by subsequent owners.^67^ Today the portrait of Cardinal Wolsey from the Weston set is in the collection of the NPG. It is easy to assert that Parsons' treatment of the structural and decorative condition of the Knole set does not correspond to approaches taken today. However, his changes to their appearance, from unacceptable decorative fragments to smartly framed paintings, has helped to ensure their survival.

## Addition of four royal portraits

**Figure 6.**
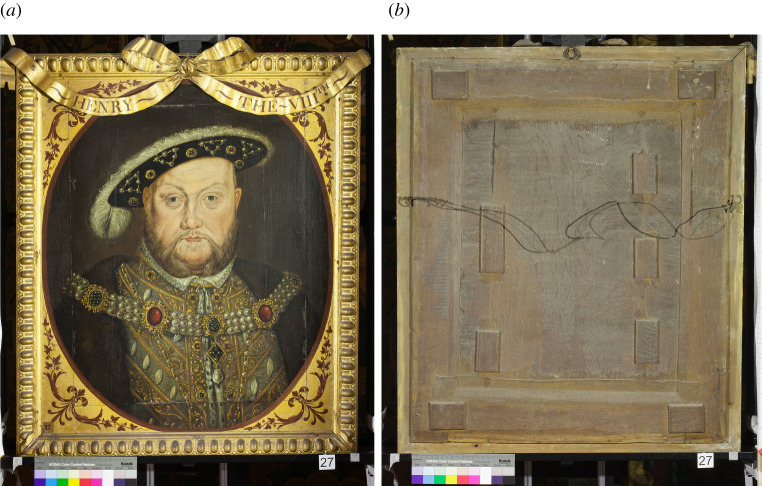
Front (*a*) and back (*b*) of the portrait of King Henry VIII (1491–1547), after Hans Holbein the younger, British (English) School (around first half of 1600s, and 1793 (extended and framed)), painting 129753 and frame 131153, 840 × 730 × 60 mm. Copyright © National Trust/Diana Jaskierny. (Online version in colour.)

Six paintings from the Knole set are now classified as non-original. They are thought to have been part of Parsons' restoration campaign in 1793, thus integrating them into the original portrait set. These are the four royal portraits of *Henry VIII* (figure 6), *Mary I*,^68^ *Elizabeth I*^69^ and *James VI & I*^70^ as well as the portraits of *George Clifford, 3rd Earl of Cumberland*,^71^ and *Admiral Blake*.^72^ The royal portraits do not appear out of place in the Knole set as many Elizabethan portrait sets featured royal sitters, for example the NPG set mentioned above.

The portraits of *Henry*, *Mary* and *Elizabeth* stylistically date from the first half of the seventeenth century.^73^ They are painted on oak panels but have been enlarged on all sides to match the dimensions of the Knole portrait set. Owing to these additions the end grain of their original wooden panels was not accessible to allow for dendrochronology.^74^ It has been suggested that these three portraits were adjusted by Parsons from existing paintings within the Knole collection^75^ and that he was responsible for creating replica frames for them to match the original set.^76^ The portrait of *James VI & I* is the only painting in the set on canvas; it is thought to date from 1620 to 1625 and is based on the portrait type of *James VI & I* by the artist Paul van Somer.^77^ Parsons added a replica frame and applied the spandrel decoration directly onto the canvas. He was also charged with supplying ‘one New Portrait’ of the *Earl of Cumberland*. This likely refers to the replacement of a damaged portrait as opposed to the addition of another sitter to the set, as Vertue's list from 1728 includes a portrait of the Earl.^78^ Finally, the portrait of *Admiral Blake* differs structurally and stylistically from the original set; the panels are butt-joined as opposed to tongue and groove, and the portrait has a coat of arms in the background.^79^ Unlike the portraits in the original set, paint extends beneath the replica frame of this painting and it has chipped edges; this suggests that the portrait has been cut down from a larger format and the frame added later.^80^ The ribbons, gilding and red-painted decoration of all six portraits match those of the original thirty-eight, yet these six frames have slight differences from those of the originals. This further supports the idea that Parsons adjusted these portraits at the same time as adding the ribbons to the remaining set. The addition of the royal portraits displays a desire by the 3rd Duke of Dorset to elevate the status of the set, perhaps coinciding with the overall reworking of the whole set. Although royal portraits were common in similar sets, there is no evidence that the lost paintings from the original set depicted monarchs. Nevertheless, the 3rd Duke would have been aware of many contemporary sets, then still *in situ*, and so could have considered the introduction of royal portraits as an act to elevate the status of his set.

Another royal portrait had been introduced into the Brown Gallery, an oil-on-canvas painting of *Henry V* painted just prior to Parsons' work, but this was housed in a black, reused, early seventeenth-century joiner's frame.^81^ An old inscription painted on the back of its original canvas reads ‘Henry ye 5th of England I.F.(?) Dorset 1790 A Present from William Hanbury Esq're’, and an ink-on-paper label inscription on the strainer reads ‘This is a Copy from an Original Picture in the Provost's lodgings at Eton College’. This introduction of a copy of a recognized portrait type in a reused antique frame shows the same desire to raise the status of the Gallery's portraits by surreptitiously adding to the collection.

Some time during the late nineteenth to early twentieth centuries, splits in many of the panels were further reinforced with timber buttons adhered to the reverse (figure 3*b*), and some of Parsons' battens were removed.^82^ The removal of several of Parsons' battens would likely only have been carried out because of ongoing instability; however, their present condition indicates that these later treatments also did not solve the issue. In addition there is physical evidence of several of the splits being reinforced with strips of canvas adhered to the reverse. These are seen going over the buttons, indicating that they were done after the buttons went on; these have also failed to keep the panels from moving.

**Figure 7.**
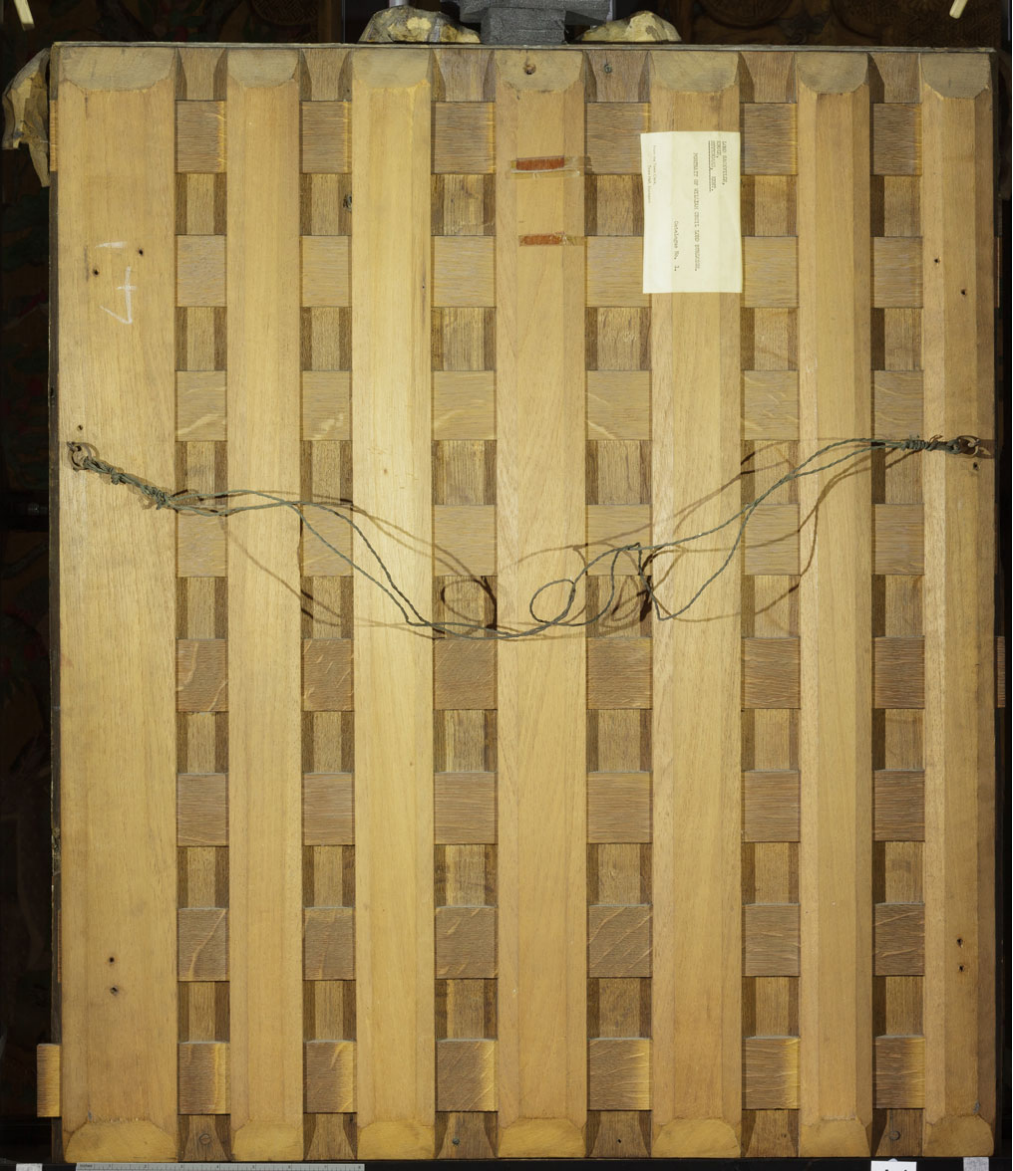
Back of portrait of William Cecil, 1st Baron Burghley, Lord Treasurer of England (1520–1598), after Marcus Geeraerts the elder, British (English) School, 1605–1608 and 1793 (repainted and embellished frame), painting 129767 and frame 131178, 850 × 700 × 60 mm. Copyright © National Trust/Diana Jaskierny. (Online version in colour.)

The portrait of *William Cecil* was singled out to have a cradle added (figure 7). This was likely to have been during the nineteenth or early twentieth centuries, and perhaps carried out by Bourtlet & Sons, who were thought to have added the buttons mentioned above. A movable hardwood cradle was attached to the reverse of the panel. A cradle is a lattice structure with wooden battens adhered at intervals along the grain direction, with a series of bridges cut in these for unfixed battens going across the grain to slot through. In theory the battens across the grain should move when the panel responds to changes in humidity, allowing lateral expansion and contraction of the panel.^83^ However, this is often not the case and cradles can result in further splitting where the panel cannot move as the horizontal battens jam. It is unclear why this particular portrait was chosen for this treatment as it is by no means more warped than most of the other thirty-seven original panels. Perhaps it was chosen as a test piece to see whether the treatment would be worth repeating on the remainder of the set. Alas, the cradle was not successful in restraining the panel and no others have any evidence they were ever cradled.

Forty of the term figures survive at Knole from the Cartoon Gallery's original scheme. Seventeen remain in the Gallery's frieze, of which only five appear possibly undisturbed. Fifteen others have been stripped of their decorative surface, refinished as dark wood, and have had their bases cut down for display in the Venetian Ambassador's Dressing Room. Seven are overpainted grey to match their redisplay as part of the panelling in the Ballroom. The term figures were made in matching pairs, which are now almost completely disrupted, making the interpretation of their specific allegorical meaning difficult. The material histories of the portrait set and term figures offer an interesting discussion when considering how differently they have been mutually redisplayed. On the one hand the portraits have been elevated, in a modern sense, from their previous category as part of a decorative scheme; they are now classified as portable easel paintings with gilt frames reminiscent of eighteenth-century high-art tastes. The terms, however, taken out of their original context, are separated, variously overpainted grey or stripped of original paint, and have therefore been demoted in meaning and status.

During the late nineteenth and early to mid-twentieth centuries, ongoing flaking of and loss from the set's decorative surface, especially over unstable areas between frame and panel, were touched up using bronze paint as a cheap substitute for gilding. This application carelessly extended onto surrounding areas of gilding. At the time it was well known that this paint, composed of base metal alloys bound in oil, darkened rapidly by oxidation.^84^ Accordingly, the gilding of most of the set is now disfigured by brown patches. This use of bronze paint demonstrates an expedient and short-term approach to the set's presentation. The desired improvement of disguising losses to the decorative surface by using a paint imitating gold leaf was only temporary, as would have been easily predicted, and fairly soon produced larger areas of distracting damage.

More recent treatment of the set during the ‘Inspired by Knole’ project sought to stabilize and ‘lift’ their appearance slightly through minimal remedial intervention. Areas of active flaking were consolidated, and distracting areas of loss to the gilding were in-painted using stable and more easily reversible watercolours. Areas of flaking in the portraits were also in-painted using reversible media. However, there was no attempt to rejoin or fill splits in the panels resulting from their original framed construction, as this would likely have fared no better than the attempts of the previous three centuries. Without removing the frames' attachment across the grain of the panels, which is considered too great an impact on their original construction and appearance, the structural issues cannot be greatly improved.

## Conclusion

The ‘Inspired by Knole’ project gave those involved the chance to evaluate the successes and failures of previous restorations. It appears that many past interventions throughout the centuries were dictated by the objects' inherent structural flaws, for example the addition of battens and buttons to prevent the splits from worsening owing to the restrictiveness of the original engaged frames. These treatments were perhaps largely futile as long as the paintings remained restrained by their engaged frames. Some restorations, principally those by Parsons, were evidently spurred on as a result of changes in tastes over time and a desire to augment the set with other paintings from the house. Parsons' alteration of the visual appearance of the set was also likely intended to mask the physical deterioration of the paint film in the spandrels and frames. Parsons' treatment was complex and technically difficult, probably involving several specialist restorers and craftsmen, but no aspect of the work was pioneering. At the time, these drastic irreversible restorations would have been more the norm. By comparison with the values and ethics that guide how conservators practise today, these interventions appear bold and damaging to the makers' original intentions. Recent efforts were undertaken between 2017 and 2019 to stabilize the paintings and frames, and focused on consolidating flaking within Parsons' campaign, thereby choosing to value his work as part of preserving the set today.

The addition of the six portraits may indicate a desire to unite paintings from Knole that date from the Tudor and Jacobean periods. The inclusion of the royal portraits may also have raised its status to that of similar contemporary sets. The likely replacement by Parsons of a damaged portrait of the *Earl of Cumberland* may indicate a different approach from that in the eighteenth century, when several badly deteriorated portraits from the set seem to have been discarded rather than being restored or replaced. Perhaps there was renewed interest in paintings from this period, or an atmosphere of cultivating the Knole collection by its then owner, the 3rd Duke of Dorset. He was a prolific collector of fine art and antiquities, and, much like the 1st Earl of Dorset, used his art collection to communicate his social standing, as seen, for example, in his collection of Reynolds paintings, displayed in the Reynolds Room at Knole.^85^ As well as being a patron of contemporary British art, the 3rd Duke was a prolific collector of Italian Old Master paintings.^86^ He travelled on the Grand Tour, where he would have been in good company with other members of the British aristocracy seeking to enhance their painting and sculpture collections. As well as ‘improving’ the existing collection, the 3rd Duke sought to leave his own mark on the legacy of the Knole collection through his ambitious purchases.^87^ Alongside acts of acquisition and cultivation at Knole it can be inferred that the 3rd Duke sought to renew the importance of the Knole portrait set as a record of his ancestor, by having it restored and augmented whilst also bringing it more into the eighteenth-century environment. Throughout the centuries the significance of the Knole collection has been noted by many prominent visitors to the house, notably in 1817 with the first published guide to the house and collections by John Bridgman.^88^ The creation of a long Brown Gallery in the eighteenth century as well as incorporating other paintings into the portrait set culminated in a need to address the aesthetics of the decorative elements of the highly valued set so that they better suited late eighteenth-century viewers' tastes, and appeared less like displaced fragments of a Jacobean interior scheme.

## Data Availability

This article does not have any additional data.

## Footnotes

1 Catherine Daunt, ‘Portrait sets in Tudor and Jacobean England: two volumes’, DPhil thesis, University of Sussex (2015). See http://sro.sussex.ac.uk/id/eprint/54260/ (accessed 1 February 2022).

2 Ian Tyers, ‘The tree-ring analysis of 22 of the 44 panel paintings in the Historical Portrait Set in the Brown Gallery, Sackville Collection, Knole’, Dendrochronological Consultancy Ltd Report no. 580, (2013).

3 Catherine Daunt, ‘Heroes and worthies: emerging antiquarianism and the taste for portrait sets in England’, in *Painting in Britain 1500–1630* (ed. Tarnya Cooper, Aviva Burnstock, Edward Town and Maurice Howard), pp. 366–370 (Oxford University Press, Oxford, 2015); Daunt, *op. cit.* (note 1), vol. 1, p. 174.

4 Catherine Daunt, ‘A Gallery of fame: the evidence for a Jacobean portrait frieze at Knole’, in *The National Trust historic houses and collections annual 2016* (National Trust, Swindon/Apollo, Bootle, 2016), pp. 28–33.

5 Daunt, *op. cit.* (note 1), vol. 1, p. 166.

6 Gerry Alabone and Emma Slocombe, ‘The Knole Cartoon Gallery’, in *Arts, buildings, collections bulletin. Summer 2017* (National Trust, Swindon, 2017) pp. 9–12. The original Cartoons by Raphael, after which the Knole versions were painted, are on long-term loan to the Victoria & Albert Museum from the Royal Collection.

7 John Bridgman, *An historical and topographical sketch of Knole in Kent; with a brief genealogy of the Sackville family* (Lindsell, London, 1817), p. 18, cited in Daunt, *op. cit.* (note 1), vol. 1, p. 143. Charles, 6th Earl of Dorset (1638–1706), sold Copt Hall in 1701, and much of its contents, including the Cartoons, were taken to Knole; Robert Sackville-West, *Knole* (National Trust, Swindon, 1998), pp. 73–74.

8 Records show that flower paintings, thought to be the same ones hanging today in the Cartoon Gallery, were brought from Kensington Palace in 1696. Melanie Caldwell, ‘Grotesque Painting Scheme, Cartoon Gallery and summary of recent research relating to the decorative scheme and restoration campaigns’ (2015).

9 Simon, Jacob, *National Portrait Gallery*, *A Guide to Picture Frames at Knole, Kent*, 1998, revised 2013, https://www.npg.org.uk/research/programmes/the-art-of-the-picture-frame/guides-knole.php (accessed 4 March 2021).

10 Ruth Jongsma, ‘Knole Cartoon Gallery: architectural paint research’ (1998).

11 Daunt, ‘Heroes and worthies', *op. cit.* (note 3), p. 366.

12 Dorian Church, ‘The Cartoon Gallery, Knole’, MA dissertation, Courtauld Institute of Art (1998). Alden Gregory, ‘Knole: an architectural and social history of the Archbishop of Canterbury's house, 1456–1538’, DPhil thesis, University of Sussex (2011). Nathalie Cohen and Frances Parton, *Knole revealed: archaeology and discovery at a great country house* (National Trust, Swindon, 2019).

13 Olivia Stoddart, ‘A technical investigation into the materials and techniques of the portrait set currently hanging in the Brown Gallery at Knole House, Sevenoaks’, Final Year Project, Courtauld Institute of Art Conservation and Technology Department (2019), pp. 42–47.

14 Daunt, *op. cit.* (note 1), vol. 1, pp. 137 and 139.

15 Recent research has disputed whether Isaacson was solely responsible for the decorative scheme in the Cartoon Gallery and Great Staircase; Town noted in his thesis that Ralph Treswell may have been present at Knole during the period of Sackville's renovation (p. 192); Edward Town, ‘A house “re-edified”: Thomas Sackville and the transformation of Knole 1605–1608’, DPhil thesis, University of Sussex (2010). Alexandra Lawson, ‘“For Delights Sake”: an investigation into the materials and techniques of those grotesque paintings on canvas in the Cartoon Gallery at Knole House’, Final Year Project, Courtauld Institute of Art Conservation and Technology Department (2018), pp. 14 and 133 (Appendix 3: Inventory 4).

16 Tyers, *op. cit.* (note 2).

17 Cerys Fry, ‘An investigation into the materials and techniques of the decorative scheme in the Cartoon Gallery at Knole House, with particular emphasis on the grotesque paintings on panel and their relationship to other elements of the scheme’, Courtauld Institute of Art Conservation and Technology Department (2017).

18 Stoddart, *op. cit.* (note 13).

19 ‘Charles III, Duke of Bourbon’ (1490–1527), 1605–1608 and 1793 (repainted and embellished frame), painting 129791 and frame 131204.

20 X-rays from Stoddart's project; Stoddart, *op. cit.* (note 18), and Daunt, *op. cit*. (note 1).

21 ‘William Cecil, 1st Baron Burghley’ (1520–1598), 1605–1608 and 1793 (repainted and embellished frame), painting 129767 and frame 131178.

22 ‘Sir Francis Walsingham’ (1532–1590), 1605–1608 and 1793 (repainted and embellished frame), painting 129771 and frame 131182.

23 NPG 32, ‘Thomas Wolsey’, unknown artist, oil on panel, 1589–1595, based on a work of *ca* 1520.

24 Daunt, *op. cit.* (note 1), vol. 1, p. 138.

25 Fry, *op. cit.* (note 17), p. 39.

26 ‘Charles Howard, 1st Earl of Nottingham KG’ (1536–1624), 1605–1608 and 1793 (repainted and embellished frame), painting 129775 and frame 131186.

27 Scanning electron microscopy with energy dispersive X-ray analysis; this analytical technique can be used to identify non-organic elements within paint films.

28 For other elements, such as the grotesques on panel and canvas, research was done at Courtauld Institute of Art Conservation and Technology Department; Lawson, *op. cit.* (note 15), and Fry, *op. cit.* (note 17).

29 X-ray fluorescence is a non-invasive method for investigation into the non-organic elements within a targeted area of a painting.

30 Jessica David and Richard Clark, ‘2021.Knole.1' XRF report on three portraits of the Knole set and one Term figure (Institute for the Preservation of Cultural Heritage, Yale, 2019), pp. 3–8.

31 Daunt, *op. cit.* (note 4), p. 32.

32 Relative humidity is the amount of moisture in air measured as a percentage of what air can hold at a given temperature.

33 Daunt, *op. cit.* (note 1), vol. 1, pp. 142–143.

34 Kent History and Library Centre, Maidstone, U269/E/79/2, *An Inventory of the Goo[ds of the] Right Honorable the Earl of Dorse[t] the 14th day of October 1706*, fol. 5r.

35 Britannica, The Editors of Encyclopaedia. “George Vertue”. Encyclopedia Britannica, https://www.britannica.com/biography/George-Vertue (accessed 1 February 2022).

36 Vertue, George, ‘Vertue II’, *Vertue note books*, vol. 2 (Oxford University Press, Oxford, 1929–1950), pp. 50–51.

37 Daunt, *op. cit.* (note 1), vol. 2, Table 9, p. 235.

38 Daunt, *op. cit.* (note 1), vol. 1, p. 142.

39 Daunt, *op. cit.* (note 1), vol. 2, Table 9, p. 235.

40 Jenny Rose, ‘An investigation into the domestic care of paintings in English country houses in the eighteenth and nineteenth centuries’, in *Studies in the history of painting restoration* (ed. Christine Sitwell and Sarah Staniforth), pp. 139–140 (Archetype, London, 1998).

41 Marcin Strojecki, Michał Łukomski, Leszek Krzemień, Joanna Sobczyk and Łukasz Bratasz, ‘Acoustic emission monitoring of an eighteenth-century wardrobe to support a strategy for indoor climate management’, *Stud. Conserv.* **59**, 225–232 (2014), at p. 232.

42 Diana Jaskierny, ‘The classification and categorisation of crack patterns and delamination found on panels in the Brown Gallery of Knole House’, Final Year Project, Courtauld Institute of Art Conservation and Technology Department, (2017), pp. 33–34.

43 Smalt was widely used by artists from the fifteenth until the mid-eighteenth century. The presence of smalt does not rule out that it was a part of the original layer structure in 1608. Duoma, Michael, *Pigments through the Ages*, http://www.webexhibits.org/pigments/indiv/history/smalt.html (accessed 1 February 2022).

44 ‘Henri de Montmorency, Constable of France’ (1534–1614), 1605–1608 and 1793 (repainted and embellished frame), painting 129794 and frame 131207.

45 Simon, *op. cit.* (note 9).

46 Duoma, *op. cit.* (note 43).

47 Simon, Jacob, *British picture restorers, 1600-1950- P*, last updated September 2021, https://www.npg.org.uk/research/programmes/directory-of-british-picture-restorers/british-picture-restorers-1600-1950-p (accessed 1 February 2022).

48 *James Brindley*, Francis Parsons, oil on canvas, 1970, 51 1/4 in. × 39 1/4 in. (1302 mm × 997 mm), NPG 6170.

49 Simon, Jacob, *British picture restorers, 1600-1950- S*, last updated September 2021, https://www.npg.org.uk/research/programmes/directory-of-british-picture-restorers/british-picture-restorers-1600-1950-s (accessed 1 February 2022).

50 National Portrait Gallery, A Guide to Picture Frames at Knole, Kent, https://www.npg.org.uk/research/programmes/directory-of-british-picture-restorers/british-picture-restorers-1600-1950-s (accessed 29 July 2021).

51 Simon, *op. cit.* (note 9).

52 Jaskierny, *op. cit.* (note 42), p. 56.

53 Britta New, ‘The painted support: properties and behaviour of wood’, in *The conservation of panel paintings and related objects: research agenda 2014–2020* (ed. Nico Kos and Paul van Duin), p. 44 (NWO, Netherlands Organisation for Scientific Research, The Hague, 2014).

54
*Ibid.*

55 As demonstrated by the treatment of *Anne* *Boleyn*, Unknown artist, oil on panel, late sixteenth century, NPG 668. Hinde, Laura, and Bolland, Charlotte, *Conservation treatment of a portrait of Anne Boleyn*, https://www.npg.org.uk/research/programmes/making-art-in-tudor-britain/case-studies/conservation-treatment-of-a-portrait-of-anne-boleyn (accessed 1 February 2022).

56 Stoddart, *op. cit.* (note 13), Appendix F.d, pp. 175–198; Jaskierny, *op. cit.* (note 42), pp. 126–134.

57 Jaskierny, *op. cit.* (note 42), p. 131; Stoddart, *op. cit.* (note 18), p. 203.

58 William I, Prince of Orange, ‘William the Silent’ (1533-1584), British (English) School, 1605-1608 and 1793 (repainted and embellished frame), painting 129760 and frame 131171, dimensions 850 × 745 × 65 mm.

59 This theory is based on visual examination by those working closely with the objects over the past five years.

60 However, the portrait of ‘John Wyclif’ may have been cut down by Parsons to match the width of others in the set, as it had been made for a uniquely wide space in the Cartoon Gallery frieze.

61 Hélène Dubois, ‘When, by whom and why? Decisive material and optical alterations of the Ghent altarpiece’, in *Van Eyck: an optical revolution* (ed. Maximilian Martens, Till-Holger Borchert, Jan Dumolyn, Johan de Smet and Frederica van Dam), pp. 236–257 (Hannibal–MSK, Ghent, 2020).

62 Anne-Sophie Augustyniak *et al., Behandeling van het Lam Godsveelluik. Verslag van fase 1. Het gesloten altaarstuk (2012-2016)*, p. 95 (Brussels, 2017).

63 Gerry Alabone and Maria Sanchez Carvajal, ‘The removal of bronze paint repairs from overgilded picture frames using an erbium:YAG laser’, *J. Inst. Conserv.* **43**, 107–121.

64 Simon, *op. cit.* (note 9).

65 NPG 4980 (1–16), oil on panel. Also known widely as the Hornby Castle set.

66 Daunt, *op. cit.* (note 1), vol. 1, p. 119.

67 *Ibid*., p. 134–135.

68 *Queen Mary I (Mary Tudor) (1516-1558)*, (after Hans Eworth), British (English) School, around first half of 1600s, and 1793 (extended and framed), painting 129761 and frame 131172, dimensions 850 × 700 × 70 mm.

69 *Queen Elizabeth I (1533-1603)*, British (English) School, around first half of 1600s, and 1793 (extended and framed), painting 129769 and frame 131180, dimensions 810 × 710 × 70 mm.

70 *King James I (James VI of Scotland) (1566-1625)*, (after Cornelius Johnson), British (English) School, around first half of 1600s, and 1793 (extended and framed), painting 129783 and frame 131196, dimensions 850 × 750 × 70 mm.

71 *George Clifford, 3rd Earl of Cumberland KG (1558-1605)*, British (English) School, 1793, painting 129772 and frame 131183, dimensions 830 × 645 × 60 mm.

72 *A Commander, possibly Lord William Howard (1593-1640)*, previously misidentified as Admiral Robert Blake (1599-1657), British (English) School, 1793, painting 129788 and frame 131201, dimensions 840 × 710 × 70 mm.

73 Daunt, *op. cit.* (note 1), vol. 1, p. 148.

74 *Ibid*.

75 Alastair Laing, ‘Knole: a series of historical portraits’, in ‘Notes on collections: Knole’ (1998), pp. 4–6.

76 Daunt, *op. cit.* (note 1), vol. 1, p. 147.

77 Roy Strong, *Tudor and Jacobean portraits* (HMSO, London, 1969), vol. I, pp. 178–180.

78 Daunt, *op. cit.* (note 1), vol. 1, p. 149.

79 The *terminus post quem* of the oak is *ca* 1610, after Sackville's commission date of 1608. Tyers, *op. cit.* (note 2), p. 52, cited in Daunt, *op. cit.* (note 1), vol. 1, p. 149.

80 Tyers, *op. cit.* (note 2), p. 52.

81 ‘King Henry V’ (1387–1422), 1780, painting 129748 and frame 131164, sight size 562 × 440 mm.

82 Jaskierny has suggested that the buttons on the reverse were added between 1890 and 1957, likely by the restorers Bourtlet & Sons Ltd, who ran a studio on Nassau Street during this period; Jaskierny, *op. cit.* (note 42), p. 56.

83 Paul Ackroyd, ‘The structural conservation of paintings on wooden panel supports’, *The conservation of easel paintings* (ed. Joyce Hill Stoner and Rebecca Rushfield), p. 462 (Routledge, Abingdon, 2012).

84 Alabone and Sanchez Carvajal, *op. cit.* (note 63), pp. 107–121.

85 John Chu, ‘High art and high stakes: the 3rd Duke of Dorset's gamble on Reynolds’, *Br. Art Stud.* Issue 2 (2016), https://doi.org/10.17658/issn.2058-5462/issue-02/jchu (accessed 4 March 2021).

86 Edward Town, *op. cit.* (note 15), p. 4.

87 Ann Bramley, ‘A Duke on the Grand Tour: John Frederick Sackville, 3rd Duke of Dorset’, *Br. Art J.* **7**, 75–81 (2006), at p. 75.

88 Bridgman, *op. cit.* (note 7).

